# Design of the PRINCESS trial: pre-hospital resuscitation intra-nasal cooling effectiveness survival study (PRINCESS)

**DOI:** 10.1186/1471-227X-13-21

**Published:** 2013-11-25

**Authors:** Per Nordberg, Fabio Silvio Taccone, Maaret Castren, Anatolij Truhlár, Didier Desruelles, Sune Forsberg, Jacob Hollenberg, Jean-Louis Vincent, Leif Svensoon

**Affiliations:** 1 Department of Clinical Science and education, Karolinska Institutet, Södersjukhuset, Stockholm, Sweden; 2 Department of Cardiology, Karolinska Institutet, Södersjukhuset, Stockholm, Sweden; 3 Department of Intensive Care, Hopital Erasme, Université Libre de Bruxelles (ULB), Route de Lennik, 808, Bruxelles 1070, Belgium; 4 Section of Emergency Medicine, Karolinska Institutet, Södersjukhuset, Stockholm, Sweden; 5 Emergency Medical Services of the Hradec Kralove Region, University Hospital Hradec Kralove, Hradec Kralove, Czech Republic; 6 Department of Anaesthesiology and Intensive Care Medicine, Charles University Prague, Prague, Czech Republic; 7 Department of Emergency Medicine, Gasthuisberg University Hospital, Leuven, Belgium; 8 Faculty of Medicine Hradec Kralove, University Hospital Hradec Kralove, Hradec Kralove, Czech Republic

**Keywords:** Cardiac arrest, Intra-arrest, Hypothermia, Outcome, Randomized clinical trial

## Abstract

**Background:**

Therapeutic hypothermia (TH, 32-34°C) has been shown to improve neurological outcome in comatose survivors of out-of-hospital cardiac arrest (OHCA) with ventricular tachycardia or fibrillation. Earlier initiation of TH may increase the beneficial effects. Experimental studies have suggested that starting TH during cardiopulmonary resuscitation (CPR) may further enhance its neuroprotective effects. The aim of this study was to evaluate whether intra-arrest TH (IATH), initiated in the field with trans nasal evaporative cooling (TNEC), would provide outcome benefits when compared to standard of care in patients being resuscitated from OHCA.

**Methods/design:**

We describe the methodology of a multi-centre, randomized, controlled trial comparing IATH delivered through TNEC device (Rhinochill, Benechill Inc., San Diego, CA, USA) during CPR to standard treatment, including TH initiated after hospital admission. The primary outcome is neurological intact survival defined as cerebral performance category 1–2 at 90 days among those patients who are admitted to the hospital. Secondary outcomes include survival at 90 days, proportion of patients achieving a return to spontaneous circulation (ROSC), the proportion of patients admitted alive to the hospital and the proportion of patients achieving target temperature (<34°C) within the first 4 hours since CA.

**Discussion:**

This ongoing trial will assess the impact of IATH with TNEC, which may be able to rapidly induce brain cooling and have fewer side effects than other methods, such as cold fluid infusion. If this intervention is found to improve neurological outcome, its early use in the pre-hospital setting will be considered as an early neuro-protective strategy in OHCA.

**Trial registration:**

NCT01400373.

## Background

Sudden cardiac death is one of the major health issues of the industrialized world [1]. Despite decades of efforts to promote cardiopulmonary resuscitation (CPR) education and the introduction of automated external defibrillators, less than 50% of cardiac arrest (CA) victims achieve a return of spontaneous circulation (ROSC) and this percentage drops to 20% or less for those patients that live in rural areas or do not have an initial rhythm that can be defibrillated (e.g., pulseless electrical activity, PEA, and asystole) [2-4]. Even fewer of these patients are alive on hospital admission and most of them will eventually die because of extended post-anoxic brain injury [5-8].

In 2002, two randomized clinical trials demonstrated the benefit of therapeutic hypothermia (TH) on neurologically intact survival in patients who were cooled in-hospital for 12 to 24 hours to 32-34°C within few hours from ROSC following an out-of-hospital cardiac arrest (OHCA) with ventricular fibrillation (VF) or ventricular tachycardia (VT) as first rhythm [9,10]. Based on these studies, the International Liaison Committee on Resuscitation have recommended the use of TH as a routine treatment of OHCA patients with VF/VT as first rhythm during post-resuscitation care. Despite the absence of randomised trials, current guidelines recommend that TH should be considered also in patients presenting with non-shockable rhythms [11]. In addition, these guidelines stated that patients resuscitated from OHCA should be cooled as soon as possible; however, the optimal timing to initiate TH in this setting remains unknown.

Several clinical studies have assessed the impact of early cooling using cold saline infusion shortly after return of spontaneous circulation (ROSC). In 2007, Kim et al. performed a feasibility study in 123 patients, which demonstrated that early use of cold fluids was safe and that the core temperature could be lowered 1.2 degrees upon arrival to hospital. Also, there was a trend towards increased survival to discharge in patients with VF/VT [8]. Recently Bernard et al. published a randomized trial on post-ROSC early cooling with cold fluids compared to in-hospital TH in 234 VF-patients. Rapid cooling decreased core temperature of patients at hospital arrival but did not improve outcome at hospital discharge compared with cooling commenced in the hospital [12].

Recent data suggested that initiating TH during CPR, before the reperfusion phase, would provide additional beneficial effects against post-anoxic brain injury. Animal studies have shown that cooling induced during CPR and prior to ROSC, the so-called “intra-arrest hypothermia” (IATH), improved neurological outcome when compared to animals that are cooled post-ROSC [13-17]. To date, there are no sufficiently large human studies that have evaluated the effects IATH compared to standard TH initiated in-hospital on neurological outcome and mortality [18]. In a retrospective analysis of 542 patients where 208 were treated with intra-arrest cold intravenous fluids, the use of IATH associated with improved ROSC rate but not with increased overall survival to hospital discharge [19]. Importantly, only 13% of the whole cohort received in-hospital TH.

Nevertheless, not all methods to induce IATH may produce the same effects on brain recovery after CA. In animal studies, global IATH has been induced by various techniques/devices, including ice packs, intravascular catheters, cold metal plates or total lung ventilation with perfluorocarbons (PFC). Moreover, some devices may selectively cool the brain primarily with less effect on core body temperature and thus more rapidly protect cerebral cells from ischemia/reperfusion injury. Only three experimental studies have compared different IATH techniques on specific outcomes [20-22]. Interestingly, cardiac function was significantly improved by IATH induced by an intravascular system compared to cold fluids [20]; in this study, IATH using cold fluids resulted in a lower coronary perfusion pressure, a greater need for epinephrine and a longer duration of CPR. In another study, PFC-total lung ventilation achieved more easily ROSC than intra-arrest cold fluids [21]. Finally, in a model of prolonged CA, trans-nasal evaporative cooling (TNEC) initiated during CPR improved the success of resuscitation compared with IATH induced by cold fluids and reduced the duration of CPR [18]. These studies supported the concept that IATH induced by cold fluids may have some deleterious effects on cardiac perfusion during CPR and may not as effective as other techniques to reduce brain injury and improve survival after CA.

TNEC is a method that has been developed to rapidly induce IATH and achieve hypothermia for human CA. The technique takes advantage of the nose as a natural orifice into the head to overcome the obstacle of cooling through the skull. The upper nasal pathways provide a large diffuse surface area and vascularity that is in close proximity to the cerebral circulation. Cooling in the nasopharynx also offers the ability to cool via direct conductive mechanisms that do not rely on spontaneous circulation [22]. In the only randomized study using such approach to induce IATH [23], 200 OHCA patients were randomized during CPR, irrespective of their rhythm, to receive either TNEC or standard of care (including post-ROSC cooling at hospital). This feasibility study showed that the method was safe for pre-hospital use. Overall survival rates were similar in the two groups (15% vs. 13%). Among patients admitted to the hospital, overall survival to hospital discharge was improved, although not significantly, during IATH (44% vs. 31%, p = 0.16). In the post-hoc analysis, the sub-group of patients with time to CPR less than 10 minutes had an increased survival rate when treated by IATH (56% vs. 29%, p = 0.04). Also, TNEC increased, although not significantly (p = 0.14), the intact neurological outcome rate from 21% to 34% when compared to the control group; these beneficial effects were more pronounced in the subgroup of patients with time to CPR less than 10 minutes (43% vs. 17%, p = 0.03).

Given these supportive data, we are conducting a randomized controlled trial comparing TNEC to conventional post-ROSC cooling. The aim is to determine whether TNEC used during CPR would improve neurological outcome in patients being resuscitated from OHCA and with a time to CPR less than 15 minutes.

## Methods/design

### Study design

This is a prospective, multi-centre, randomized, single-blind, controlled study conducted by the emergency responders of different emergency medical systems (EMS). In the treatment arm, patients will be immediately cooled by paramedics and/or emergency physicians using the RhinoChill device. Intra-arrest cooling will be continued after ROSC and until hospital admission for patients who will be resuscitated while it will be interrupted at the end of CPR in case of unsuccessful resuscitation. In the control arm, patients will receive standard of care and will be treated with TH after arrival at the hospital.

### Study sites

The sites including patients in this study are: a) Karolinska Institutet (Stockholm, Sweden), defined as the Principal Investigator (PI); b) Hôpital Erasme (Brussels, Belgium); c) Universiteit Ziekenhuis Leuven (Leuven, Belgium); d) Emergency Medical Services of the Hradec Kralove Region (Hradec Kraklove, Czech Republic). In Belgium and Czech Republic, physicians will enroll patients while in Sweden both physicians and paramedics will recruit patients.

### Inclusion/exclusion criteria

All victims of CA will be screened for study eligibility upon arrival of the first responding team. Inclusion criteria are: age ≥ 18 years; witnessed (heard or seen) collapse; not having a pulse. Exclusion criteria are: age ≥ 80 years; etiology of cardiac arrest due to trauma, severe bleeding, drug overdose, cerebrovascular accident, drowning, smoke inhalation, electrocution, hanging; already hypothermic (≤ 34°C at tympanic or rectal temperature); an obvious barrier to placing intra-nasal catheters (i.e., intranasal obstruction); Do Not Attempt to Resuscitate (DNAR) orders or terminal disease; known or clinically apparent pregnancy; known coagulopathy (except therapeutically induced); chronic oxygen therapy at home; response time (call to first EMS CPR) > 15 minutes.

### Randomization

Randomization will be carried out in blocks of four and each site will be given sets of sequentially numbered envelopes with randomization assignments provided in a 1:1 manner to distribute to the participating pre-hospital vehicles. Individual envelopes will be placed in each RhinoChill pack at the time of site initiation, and replaced as patients are enrolled. The RhinoChill pack will be carried to every potential subject, and the envelope will be opened once the subject has been qualified as meeting all inclusion and exclusion criteria. Neither EMS or hospital personnel will be blinded to treatment, since the control patients are easily distinguishable from patients undergoing device placement and nasal cooling. However, medical personnel making the final neurological assessment of the patient prior to discharge will be blinded to patient’s group assignment.

### Study treatment

The resuscitation attempt will follow current guidelines [24] to the greatest extent possible. After airway management (i.e. laryngeal mask or intubation), patients are randomized to early cooling or standard care. In patients randomized to early cooling, the RhinoChill catheters should be placed and cooling initiated immediately. To place the nasal catheters and start cooling takes approximately 1 minute. Cooling should be performed with the oxygen flow set to 40 L/min. There should be no changes to the care received by patients randomized to the control arm. Resuscitation attempts should be continued for at least 30 minutes after EMS arrive on the scene in all patients before deciding that further interventions are futile.

### Device description

The RhinoChill works by spraying a liquid coolant onto the upper surface of the nasal cavity, where it evaporates and absorbs heat from the tissue, thereby cooling the tissue and the innate vasculature that supplies blood to the brain. The coolant is an inert liquid (perfluorohexane, PFH) at one atmosphere of pressure and can carry 20 times more oxygen than saline. It has a surface tension that is lower than water so it will spread uniformly and quickly throughout the space in which it is sprayed. Oxygen or air is delivered with the liquid coolant to maximize its evaporation. Medical grade oxygen or breathing air with a supply pressure of 60 psi and sufficient quantity to provide a 40 L/min flow rate over the treatment period is required in order to operate the RhinoChill.

The coolant vapor, along with the gas, escapes the nasal cavity through the nostrils or the mouth. In the event that all the coolant is not evaporated, it is possible that it will either trickle out of the nostrils or trickle down the pharynx into the mouth or stomach. Because the coolant is immiscible in water, it is not absorbed in any significant quantity into the body. The minute quantities that may be absorbed into the blood or inhaled into the lungs are expired through the lungs in a relatively short period. Local temperatures within the nasal cavity are expected to cool to around 2°C.

The RhinoChill consists of three components: the tubing set, the control unit, and the coolant bottle. The tubing set is a single-use device that delivers the pressurized gas and coolant mixture to the patient. The proximal end attaches to the control unit to which a medical gas source is connected. Distal to the control unit is the interface for the coolant bottle; this consists of a dip tube connected to a bottle interface collar into which is incorporated a liquid flow indicator. Liquid coolant is driven out of the bottle by the pressurized gas, through a 0.22 μm filter, and then the gas and coolant are delivered to the nasal catheters. The trans-nasal catheters are joined together with a hub at the proximal ends; the catheters are mated to the gas and liquid delivery lines via an integral manifold. The catheters have separate gas and liquid capillaries that converge at each of 12 spray ports along the dorsal surface of the catheter. Close contact of the liquid PFH with the pressurized gas at each of the spray ports results in efficient nebulization of the vapor from each of these ports. Each catheter also has three pressure sensing ports along the ventral surface of the catheter that transmit the local pressure in the nasal cavity to the control unit. The control unit also has a mechanical over-pressure safety valve, which is designed to vent excess oxygen to prevent a pressure greater than 60 psi from entering the device.

The coolant bottle holds 1 liter of the evaporative coolant that will last 30 minutes when the oxygen or air flow rate is set to 40 L/min. The RhinoChill is configured for use in a stable hospital setting (e.g., hanging from an I.V. pole mount) or packaged in a backpack that integrates a 3 L (900 liters gas) oxygen or air bottle, and weighs approximately 9 kgs for use in the ambulance and field setting. It is expected that at least one tubing set and 1 L of coolant will be needed for each subject enrolled in the early cooling arm. Patients that are resuscitated after RhinoChill cooling is initiated will likely require 1–2 additional bottles of coolant before in-hospital cooling can be initiated.

### Post-resuscitation care

Return of spontaneous circulation will be defined as obtaining an organized rhythm and palpable pulse sustained for 20 minutes. Once an organized rhythm and palpable pulse is achieved, subjects will have their temperature taken via the tympanic route, stabilized and then be transported to the hospital. If a patient wakes up after ROSC is achieved, cooling will be discontinued; on the opposite, subjects will, if needed be intravenously administered bolus doses of sedation and analgesia for transport to the hospital. Doses of sedation and analgesia will be dictated by the institutional standard cooling protocol. The oxygen supply in the transport vehicle should be used to continue RhinoChill cooling during transport to the hospital. Normal transport procedures will be used for patients randomized to the control arm. Infusions of cold saline or cooling with cold packs will not be permitted in the pre-hospital setting for either group. Upon hospital arrival, a systemic temperature probe will be placed (i.e., bladder, arterial, rectal) and core temperature will be recorded. Systemic hypothermia on arrival at Intensive Care Unit (ICU) for both patient groups (treatment and control) will be performed as follows:

–Duration of hypothermia for at least 24 hours.

–Target temperature 33°C ± 1°C.

–Recording of core- and tympanic temperature every 15 minute during the induction period (i.e., until target temperature is reached).

–Rewarming rate at 0.2-0.5°C / hour.

–Control of post-cooling hyperthermia.

Subjects randomized to the early cooling group with the RhinoChill shall continue being cooled with the RhinoChill until systemic cooling procedures can be started. Intravenous sedation, analgesia and neuromuscular blockade (if used) should be initiated upon hospital arrival according to institutional cooling protocols. Doses should be adjusted as necessary over the course of cooling/re-warming. After the subject has been prepared with the standard hypothermia device, the RhinoChill should be turned off, but the intranasal catheters should be left in place while transitioning the subject to the standard hypothermia protocol.

Intermittent activation of the RhinoChill may be considered if the core temperature does not continue to drop via the systemic cooling method. Cooling via the RhinoChill system will be halted immediately if any adverse event related to the use of the RhinoChill develops.

For those patients in the control group, the standard cooling procedure will be started as soon as practical. Intravenous sedation, analgesia and neuromuscular blockade (if used) should be initiated upon hospital arrival according to institutional cooling protocols. Doses should be adjusted as necessary over the course of cooling/re-warming. Temperatures (tympanic and core) will be monitored periodically over the first 48 hours after hospital arrival for all patients. ECG, peripheral oxygen saturation, heart rate and blood pressure monitoring will continue throughout the acute care period. Ventilation settings should be adjusted to maintain arterial oxygen saturation 94-98% (e.g., PaO2 80–120 mmHg) and normocapnia (e.g., PaCO2 35-45 mmHg). A positive end-expiratory pressure (PEEP) level should be adjusted to reduce FiO2 below 60%, whenever possible. Electrolytes will be substituted as necessary to maintain normal ranges. Blood pressure should be kept above 65 mmHg at all times. Drops in pressure should be treated primarily with crystalloid fluids. If sufficient pressure control cannot be achieved with fluids alone, vasopressor drugs, such as adrenaline or norepinephrine, should be used. Serum blood glucose should be kept between 110 and 150 mg/dl with insulin administration. Enteral feeding will be initiated according to local guidelines.

### Data collection

Data collection concerning the cardiac arrest will include time to collapse; bystander CPR; location; time of arrival of basic and advanced life support, ALS, teams; time of emergency call; time of first emergency medical team initiated CPR, patient characteristics (i.e. gender, weight, age, comorbidities), resuscitation effort (time CPR is initiated by the first emergency medical staff, EMS, responder; time cooling initiated - if cooled; mechanical CPR, if not manual; time intravenous access obtained; first rhythm; total adrenaline/epinephrine dose; shock time, strength, and success; additional medication(s) and dose(s); time airway is secured; recurrent VF or re-arrest; time randomized; time ROSC or death declared). Also, quality of CPR, including the number and depth of chest compressions/min as well as CPR hands-off time, will be recorded whenever possible. After hospital admission, tympanic temperature; heart rate, peripheral oxygen saturation (SpO2), mean arterial pressure, end-tidal carbon dioxide (EtCO2), arterial blood gas analysis, initial ECG findings and Glasgow coma score will be recorded. Concomitant interventions (e.g., coronary angiogram, aortic balloon pump, bypass surgery, internal cardiac defibrillator placement) will also be recorded. The systemic cooling procedures, including the system used, the core temperature location, the duration and the hypothermia reversal algorithm will be recorded.

The Steering Committee has the responsibility to perform periodic and spot checks visits to monitor the progress of the clinical study. Completed Case Report Forms (CRFs) will be reviewed for completeness, compliance with the investigation plan, and appropriate device use and accountability. Case Report Forms will be provided to each site for each subject enrolled in the study. Required data concerning patient treatment and test results will be recorded on the CRFs at the time of the procedure or as soon as possible thereafter. Information recorded in the CRFs will be corroborated by data in the subject’s medical records. Completed and monitored CRFs will be uploaded on a website developed by the PI, which will be overseeing data entry and data quality management. Data on safety will be provided to the Steering Committee with regular time intervals.

The Steering Committee will review study integrity, safety and risk/benefit issues at periodic intervals throughout the study. The frequency of these reviews will be dependent upon the rate of patient enrollment and relevant safety issues. Independent analyses of serious adverse events will be performed and adjudicated if the frequency or nature of serious adverse events warrants it.

### Follow-up

Subjects treated with the RhinoChill will undergo rhinoscopic examination of the nasal cavity if it is warranted based on clinical signs of intra-nasal trauma (e.g., bleeding from the nostrils, whitening of the nose or other treatment-related observations). All patients still alive will undergo a neurological examination 72 hours after hospital admission. Neurological status will be classified using the Cerebral Performance Category (CPC) [25]. Subjects will be followed until death or at 90 days. The following data will be recorded concerning their hospital course: date/time subject wakes; date/time subject is taken off ventilator; date/time subject is discharged from ICU; date/time subject dies (if in hospital); date/time subject is discharged from hospital; discharge disposition; CPC at hospital discharge. CPC will be evaluated at 90 days through a telephone interview by an independent physician using the Glasgow Outcome Scale (GOS) and the 15D instrument. If there are difficulties in CPC-classification, the patient will be assessed by a neurologist at an out-clinic visit. Clinically significant serious adverse events will be recorded from the time ROSC is achieved through the earliest of the following three events: death, hospital discharge, or one week following admission. The nature and severity of the adverse event, the relationship to the RhinoChill Device, management and outcome will be recorded on the CRF.

### Study outcomes

The main objective of this study is to assess the impact on neurological intact survival rates at 90 days of trans-nasal IATH compared to standard treatment. Thus, the primary end-point will be the neurological intact survival rates (i.e. patients with CPC score of 1–2) at 90 days after arrest among all those admitted to the hospital.

Secondary end-points will include:

–Overall survival at 90 days among patients admitted to the hospital.

–Survival and neurological intact outcome at 90 days among all included patients.

–ROSC rate.

–Survival to hospital admission.

–Proportion of patients reaching target temperature (≤ 34° Celsius) within 4 hours of call to dispatcher.

–All adverse events occurring within 24 hours of beginning ALS procedures.

–Serious adverse events (SAE) from the time of patient randomization through the first seven days of hospitalization.

Importantly, in the cases where withdraw of life support is decided due to poor prognosis, the reasons must be clearly stated in the CRF, as well as results of neurological examination or additional tools (i.e. brain imaging, biomarkers, electroencephalography, somatosensory evoked potentials - SSEPs), which are used. No specific recommendation on post-anoxic coma prognostication has been provided in this study.

### Statistical analysis

The primary outcome measure of this study is neurological intact survival at 90 days among all patients admitted to the hospital. Considering data from the previous trial [23], we postulated that the using of IATH with TNEC technique could improve this primary outcome from 21% to 37%. For a 80% power and α error of 0.05, the study requires 150 patients admitted to the hospital who should be randomized in each arm: however, considering a ROSC rate around 40% and 10% of lost to follow-up, the study will require a sample size of 418 patients for each arm (836 in total), regardless of initial rhythm. Analysis of data will be based on “Intention-to-treat”. A first interim analysis will be performed after 200 patients; conditional power for meeting the primary endpoint will be, if needed, recalculated at that time, and if the interim results do not correspond to the primary end-point, termination of the study for futility will be considered. In case of sample calculation exceeding 500 patients, other potential recruiting centers will be included in the study. Other interim analyses will be performed each 200 patients included. Early stopping for efficacy reasons will only be considered if major outcome differences are seen between the groups according to the Haybittle rule with a p-value ≤ 0.001.

Stratified analyses will be performed for patients whose first recorded rhythm is VF/VT versus those in whom the first recorded rhythm is PEA/asystole. Sub-group analyses will be performed for subjects where CPR was initiated within 10 minutes by a first EMS response team or where cooling was started within 15 minutes. The study is expected to last until 2016.

### Consent/ethics

Because inclusion is decided as soon as possible after EMS arrival, informed consent cannot be obtained before enrollment into the study, but deferred consent will be obtained in each case. The RhinoChill device has received CE marking and did not raise any major safety issues in previous studies [23,26]; as such, local Ethics Committees (The Regional Ethical Review Board in Stockholm - reference study number: 2010/383-32; Comité d’Ethique de l’Hôpital Erasme – reference study number: P2012/321; Ethics Committee, University Hospital Hradec Kralove –reference study number: 201204 S24ZP; Commissie Medische Ethiek van Universitaire Ziekenhuizen KU Leuven; reference study number: S54255) approved study in eligible patients who are unable to provide consent prior to their treatment, as they will necessarily be comatose. Ethical consideration for treating subjects without their express consent is in accordance with the World Medical Association Helsinki Declaration of 1964, as revised at the 59th General Assembly in Seoul in 2008. For subjects admitted to the hospital after ROSC, their legal representative will be informed of the study as soon as possible and must approve for data collection and continuation of the study. Subjects who will show neurological recovery will be informed of their study participation and be asked to provide their consent for the use of their data.

### Data safety management

The Data Safety Monitoring Committee (DSMC) is constituted and will review data every 3 months. Given that it is expected that more than half of the patients will die at the scene or during the hospital stay, and that recurrent cardiac arrest at any time is possible with standard care, it is not considered appropriate to report every death to the DSMC as a serious adverse effect. Sites will report all SAEs that occur within 7 days of enrollment directly to DMSC. A “technical device failure” is defined as a failure of the device to perform its intended function when used in accordance with the instructions for use. Technical device failures will be recorded and evaluated for possible untoward effects on the subject. All clinically significant AEs or those that appear to be related to the use of the RhinoChill (i.e., whitening of the nose; epistaxis; mucosal irritation/dryness; para-sinus emphysema) as well as those that could potentially harm the patient (i.e., pleural effusion) will be recorded on the CRF from ROSC through the end of the cooling period.

## Discussion

The most common limitation in CA studies is the lack of either pre-hospital or in-hospital standardised protocols to manage these patients. This also applies to TH. Most studies on cooling after CA were limited to the in-hospital setting, where patients were treated after admission to the Emergency Department or ICU and evaluation of outcome is restricted to hospital discharge and corrected for several in-hospital factors [27,28]. This approach may of course create different confounders; indeed, pre-hospital factors have a significant impact on survival of resuscitated patients and the presence of different types of EMS may provide unbalanced quality of CPR and pre-hospital care that could later affect the measured outcomes [29,30]. On the other hand, pre-hospital studies generally lacked a standardised in-hospital management of these patients while post-resuscitation care can also substantially alter neurological recovery after anoxic injury [19,31]. Moreover, in some studies, cooling was also interrupted after hospital arrival, thus limiting its potential benefits [19]. These are all important issues to consider when discussing the optimal time to start TH after CA.

The importance of this trial is the comprehensive approach where the whole chain of factors and management of the patient is investigated. This is indeed the largest trial investigating a brain selective cooling approach, initiated in the pre-hospital setting, and involving numerous people in the treatment of such patients from the field until ICU admission. The rationale behind the PRINCESS study is to confirm the promising results observed in the PRINCE trial, although with the ambition to improve the management of the patients in terms of earlier initiation of cooling, limited cooling interruptions from the pre-hospital to the in-hospital setting, a standardised in-hospital management and a longer follow-up period for improved neurological evaluation. Importantly, we aimed to limit the number of centres to ensure that the use of TNEC and patients management should be as standardised as possible. The risk of having many centres with low inclusion rate and poor skills with Rhinochill use may influence the final results.

Few data are available on the optimal timing to initiate TH after CA. The PRINCESS study will test the effect of TNEC during CPR on patient neurologically intact survival when compared to standard in-hospital cooling. If brain selective cooling will improve brain recovery after anoxic injury, an important improvement in the pre-hospital management of such patients will be obtained.

## Abbreviations

ALS: Advanced life support; CA: Cardiac arrest; CPC: Cerebral performance category; CRF: Clinical report form; CPR: Cardiopulmonary resuscitation; DNAR: Do not attempt to resuscitate; EMS: Emergency medical service; etCO2: end-tidal carbon dioxide; IATH: Intra-arrest therapeutic hypothermia; ICU: Intensive care unit; OHCA: Out-of-hospital cardiac arrest; PEA: Pulseless activity; PEEP: Positive end-expiratory pressure; PI: Principal investigator; PFC: Perfluorocarbon; ROSC: Return of spontaneous circulation; SAE: Serious adverse event; SSEP: Somato-sensory evoked potential; TH: Therapeutic hypothermia; TNEC: Trans-nasal evaporative cooling; VF: Ventricular fibrillation; VT: Ventricular tachycardia.

## Competing interests

The authors declare that they have no competing interests.

## Authors’ contributions

PN, FST, MC and LS prepared this paper; PN, FST, SF, JH, MC and LS collaborated in the design of the trial and organized the initial ethics applications; MC and LS were responsible for funding and paramedic education. PN and LS are responsible for study governance and roll out of the trial. PN is responsible for logistical support of the study. AT, DD, JLV and PN are Site Investigators for Hradec Kralove, Leuven, Brussels and Stockholm, respectively. All authors contributed substantially to the design and methodology of this study and to the writing of this manuscript. All authors have read and approved the final manuscript.

## Pre-publication history

The pre-publication history for this paper can be accessed here:

http://www.biomedcentral.com/1471-227X/13/21/prepub
